# Dr. William Steer Riding

**Published:** 1911-08-26

**Authors:** 


The death is announced of Dr. William Steer Riding, at the age of seventy-five. He had been for some time living in retirement at Honiton, Devon. Dr. Riding studied in London and Edinburgh, holding the M.D. degree in the latter University since 1857 and the B.A. of London. Some of his studies were taken at King's College, of which he was an Associate. He became a member of the Royal College of Surgeons of England in 1858, and a Licentiate of the Society of Apothecaries in the same year. Dr. Riding took a large interest in entomology, and was a Fellow of the Entomological Society.

